# Unfriendly Fire: How the Tobacco Industry is Destroying the Future of Our Children

**DOI:** 10.15388/Amed.2020.28.1.6

**Published:** 2021-02-08

**Authors:** Andrew Bush, Thomas Ferkol, Algirdas Valiulis, Artur Mazur, Ivane Chkhaidze, Tamaz Maglakelidze, Sergey Sargsyan, Gevorg Boyajyan, Olga Cirstea, Svitlana Doan, Oleksandr Katilov, Valeriy Pokhylko, Leonid Dubey, Edita Poluziorovienė, Nina Prokopčiuk, Vaida Taminskienė, Arūnas Valiulis

**Affiliations:** Imperial College Centre for Paediatrics and Child Health, London, UK National Heart and Lung Institute, London, UK Royal Brompton Harefield NHS Foundation Trust, London, UK; Washington University School of Medicine, St. Louis, Missouri, USA; Vilnius University Medical Faculty Institute of Health Sciences, Vilnius, Lithuania; Medical College of Rzeszow University, Department of Pediatrics, Pediatric Endocrinology and Diabetes, Rzeszow, Poland; Tbilisi State Medical University, Department of Paediatrics, Tbilisi, Georgia Iashvili Central Children’s Hospital, Tbilisi, Georgia; Ivane Javakhishvili Tbilisi State University, Department of Pulmonology, Tbilisi, Georgia Chapidze Emergency Cardiology Center, Tbilisi, Georgia Planning Committee of Global Initiative Against Chronic Respiratory Diseases (WHO GARD), Geneva, Switzerland; Arabkir Medical Centre, Instutute of Child and Adolescent Health, Yerevan, Armenia; Arabkir Medical Centre, Instutute of Child and Adolescent Health, Yerevan, Armenia; University of Medicine and Pharmacy “Nicolae Testemitanu”, Department of Paediatrics, Chisinau, Republic of Moldova; Kyiv Medical University, Department of Public Health and Microbiology, Kyiv, Ukraine; National Pirogov Memorial Medical University, Vinnytsya, Ukraine; Ukrainian Medical Stomatological Academy, Department of Paediatrics, Poltava, Ukraine; Lviv National Medical University by Danylo Galytsky, Lviv, Ukraine; Vilnius University Medical Faculty Institute of Clinical Medicine, Vilnius, Lithuania; Vilnius University Medical Faculty Institute of Clinical Medicine, Vilnius, Lithuania; Vilnius University Medical Faculty Institute of Health Sciences, Vilnius, Lithuania; Vilnius University Medical Faculty Institute of Health Sciences, Vilnius, Lithuania Vilnius University Medical Faculty Institute of Clinical Medicine, Vilnius, Lithuania Planning Committee of Global Initiative Against Chronic Respiratory Diseases (WHO GARD), Geneva, Switzerland

**Keywords:** Acute lung injury, alveolar haemorrhage, eosinophilic pneumonia, lipoid pneumonia, innate immune system, nicotine, e-cigarettes, vaping, EVALI, children

## Abstract

Tobacco has long been known to be one of the greatest causes of morbidity and mortality in the adults, but the effects on the foetus and young children, which are lifelong, have been less well appreciated. Developing from this are electronic nicotine delivery systems or vapes, promulgated as being less harmful than tobacco. Nicotine itself is toxic to the foetus, with permanent effects on lung structure and function. Most vapes contain nicotine, but they also contain many other compounds which are inhaled and for which there are no toxicity studies. They also contain known toxic substances, whose use is banned by European Union legislation. Accelerating numbers of young people are vaping, and this does not reflect an exchange of vapes for cigarettes. The acute toxicity of e-cigarettes is greater than that of tobacco, and includes acute lung injury, pulmonary haemorrhage and eosinophilic and lipoid pneumonia. Given the worse acute toxicity, it should be impossible to be complacent about medium and long term effects of vaping. Laboratory studies have demonstrated changes in lung proteomics and the innate immune system with vaping, some but not all of which overlap with tobacco. It would be wrong to consider vapes as a weaker form of tobacco, they have their own toxicity. Children and young people are being targeted by the vaping industry (which is largely the same as the tobacco industry), including on-line, and unless an efficient legislative program is put in place, a whole new generation of nicotine addicts will result.

## Abstract (lt)

**Nedraugiška ugnis: kaip tabako pramonė griauna mūsų vaikų ateitį:**

**Santrauka. **Seniai žinoma, kad tabakas yra viena iš dažniausių suaugusiųjų sergamumo ir mirštamumo priežasčių, tačiau visam gyvenimui pėdsakus paliekantis tabako poveikis vaisiui, kūdikiui ir vaikui išnagrinėtas mažiau. Dar mažiau žinoma apie santykinai neseniai atsiradusias elektronines nikotino tiekimo sistemas – elektronines cigaretes, kurios tariamai yra mažiau kenksmingos nei „tradicinis“ tabako rūkymas. Nikotinas yra toksiškas žmogaus vaisiui – jis turi ilgalaikį poveikį plaučių augimui, struktūrinei ir funkcinei brandai. Daugumos elektroninių cigarečių sudėtyje randama nikotino, tačiau jose taip pat yra kitų įkvepiamųjų junginių, kurių naudojimas ribojamas Europos Sąjungoje, taip pat junginių, kurių toksiškumo tyrimai dar nėra iki galo atlikti. Kai kurie pokyčiai, tačiau ne visi, sutampa su „tradicinio“ tabako rūkymo sukeliamais pokyčiais. Elektroninių cigarečių ūminis toksiškumas yra didesnis nei įprastų cigarečių – tai atspindi dokumentuoti ūminio plaučių pažeidimo, kraujavimo į alveoles, taip pat eozinofilinės bei lipoidinės pneumonijos atvejai. Atsižvelgiant į didesnį ūminį toksiškumą, vidutinio ir ilgalaikio elektroninių cigarečių rūkymo poveikis kelia nerimą. Laboratoriniai tyrimai atskleidė su elektroninių cigarečių rūkymu susijusius grėsmingus plaučių proteomikos ir įgimtos imuninės sistemos pokyčius. Būtų neteisinga laikyti elektronines cigaretes lengvesne įprastų cigarečių rūšimi, nes elektroninės cigaretės pasižymi tik joms būdingu toksiškumu. Elektroninių cigarečių pramonė nusitaikė į internetu pasiekiamus vaikus ir jaunimą, rūkančių jaunų žmonių skaičius didėja sparčiausiai. Jeigu bus delsiama sukurti veiksmingą teisinio užkardymo sistemą, išaugs nauja priklausomybės nuo nikotino karta.

**Raktiniai žodžiai:** ūminis plaučių pažeidimas, kraujavimas į alveoles, eozinofilinė pneumonija, lipoidinė pneumonija, įgimta imuninė sistema, nikotinas, elektroninės cigaretės, rūkymas, EVALI, vaikai.

The tobacco industry has long understood that youths are an essential market, and advertising campaigns have targeted children and adolescents [1]. The havoc wreaked over generations has been well documented before, creating lifelong tobacco users who are at much greater risk for chronic obstructive pulmonary disease and lung cancer, leading causes of morbidity and mortality worldwide [2]. Less well appreciated, but even more important, are the effects of the tobacco on the foetus and the pre-school child. The purpose of this review is firstly to summarise the effects of tobacco, and especially nicotine, in early development, and then discuss how it relates to the latest attempts of the tobacco industry to profit from nicotine addiction, namely electronic cigarettes (e-cigarettes, a modern weapon of mass destruction) or electronic nicotine delivery systems (ENDS) [3-5]. We searched PubMed and our personal archives for relevant references, including searching the bibliographies of relevant manuscripts. We have excluded abstracts and conference proceedings. We will discuss the acute toxicity of e-cigarettes, how this differs from the effects of tobacco, and summarise the literature showing long-term effects of e-cigarettes. We will show from scientific studies the threat ecigarettes pose to youths, that these products are not merely “harmless water vapor” [6], but contain noxious and potentially harmful ingredients. Finally, we will highlight the absolute importance of a strategy to protect children and young people (CYP).

## The earliest, vulnerable days

*In utero*, the foetus is potentially exposed transplacentally to adverse exposures of the mother, which may be modulated by maternal genotype [7]. These exposures will interact with the foetal genotype, which may itself be subject to epigenetic regulation. These effects have been summarised elsewhere [8], but in summary, the outputs are any or combinations of prematurity and low birth weight (an all-too-well known effect of smoking in pregnancy [9]), altered lung structure, altered immune function, and sensitisation to the effects of adverse events in adult life, for example occupational exposures (below). Many of these effects are directly due to nicotine; but the foetal toxicity of the many other components of tobacco smoke are unknown, which should itself sound a warning.

***Antenatal effects on lung structure.*** A series of experimental studies whereby pregnant animals are exposed to nicotine, have established that an array of worrying structural changes can be produced in the developing lung. These include increased antenatal collagen deposition [10] and MUC5AC expression [11]. There is dysanaptic airway growth, whereby the foetal airways are longer than normal with reduced flows and postnatal airway hyper-responsiveness in the absence of infection and inflammation [12]. It should be noted that dysanaptic airway growth is associated with subsequent adverse outcomes in obesity asthma [13], and that very early airway hyper-responsiveness is associated with adverse long term respiratory outcomes [14-16]. There is also evidence of airway wall thickening [17] and airway instability due to loss of the alveolar tethering points on airway [18] which physiologically lead to interdependence. In terms of the lung parenchyma, there is evidence of reduced secondary septation and alveolar hypoplasia leading to early emphysema [19, 20].

***Antenatal effects on immune function.*******Cord blood studies have documented that there is an increased blood mononuclear cell proliferative response to allergen in babies of mothers who smoked in pregnancy [21]. There is also reduced cytokine and Toll-like receptor function in cord blood mononuclear cells [22]. Challengingly, babies who will go on to wheeze with rhinovirus, respiratory syncytial virus or any wheeze trigger have abnormal cytokine responses at birth, for example reduced interleukin (IL)-13 responses. The exact long term significance of many of these findings is unclear, but they give proof of concept that there is more than just an effect on lung structure from antenatal smoking.

***Sensitisation to future adverse stimuli.*** The mechanisms of these effects are unclear, but their existance is indubitable. The impairment of spirometry is much greater in children who grow up and smoke if their mother was also a smoker [23]. Childhood deprivation, including maternal smoking, is associated with accelerated decline in adult lung function and a greater risk of chronic obstructive pulmonary disease (COPD) [24]. Spirometry was measured twice in 12,282 adults, 9-11 years apart, in two cohorts, SPALDIA and ECRHS. The mean annual rate of decline in first second forced expired volume (FEV_1_) decline was ~25-30 ml in women and ~35-40 in men. FEV_1_ decline was more rapid in those whose mothers had smoked. The effects of personal smoking effects were also potentiated by maternal smoking [25]. Svanes *et al* tested the hypothesis: early life disadvantage sensitises to occupational lung disease. They asked 134,499 adults to fill in the RHINE III questionnaire on occupational cleaning. They defined early life disadvantage (defined as maternal smoking, severe respiratory tract infection in age less than five years, winter born, maternal age >35 years). In 2138 cleaners, there was an increased risk of wheeze, ‘adult onset’ asthma, and self-reported COPD. The strongest effect was with early life disadvantage [26]. In the latter two studies, the extent to which smoking contributed is unclear, but it is difficult to believe tobacco was an innocent bystander. Finally, there is at least animal evidence that exposures in critical time windows can increase the severity of influenza infection [27].

***Transgenerational effects?*** Two large studies have shown that if a grandmother is a smoker, even if her own daughter does not smoke, her daughter’s children, her grandchildren, are more likely to develop asthma [28, 29].

***Verdict at the sunset of the pre-e-cigarette era.*** There is conclusive proof, only some of which is summarised above, that nicotine is a chemical which is dangerous to the developing lung, and has adverse effects which are lifelong. Indeed, low lung function secondary to smoking is associated with early all cause morbidity and mortality, not just COPD [30-34]. Tobacco of course contains many other noxious chemicals, and the implication of nicotine cannot be taken to mean that other chemicals are not equally or even more dangerous. More worrisome, because of perceived lower health risks, e-cigarette use has increased among pregnant women [35], increasing exposure of the unborn child to nicotine and other toxic inhalants.

## What are e-cigarettes, and who manufactures them?

There are numerous different devices, some mimicking cigars, pipes and conventional cigarettes, others (JUULs) looking like a USB port, and, the height of impudence, some products actually look like asthma metered dose inhalers. Essentially, they consist of a battery, a reservoir containing the solution to be inhaled, a heating element or atomizer, and mouthpiece. The aim is to heat the liquid to produce an aerosol that is inhaled by the user. Increasingly, the industry is owned or controlled by tobacco companies, which is of itself sufficient to make anyone suspicious about motives.

The main aim of e-cigarettes is nicotine addiction, although in fairness, there are nicotine free liquids. Frequently, the absolute nicotine concentration is greater than that on the label, indicating a lack of quality control [36]. JUULs for example are designed to deliver a higher nicotine dose, presumably to maximize the likelihood of addiction. The e-liquid usually contains propylene glycol or glycerin as a solvent for the nicotine, as well as flavoring chemicals. Another major difficulty in assessing e-cigarettes is that there are literally thousands of liquids, and nowhere is it possible to find out what each of these liquids contains. For example, there were 7,764 flavour labels reported on websites in 2013-4, rising to a staggering 15,586 in 2016-7 [37]. One of the myths peddled by the industry is that these are largely colourings and flavourings, which can be eaten without harm. But eating is not the same as inhaling! Indeed, propylene glycol and flavourants have been shown to be toxic to airway epithelial cells in vitro and in vivo [38, 39].

What we do know about this tsunami of e-liquids is far from reassuring, and are not controlled or regulated in some nations [40]. Analyses of 18 different flavoured, nicotine, and no-nicotine ecigarette cartridges showed detectable levels of known carcinogens and toxic chemicals, including tobacco-specific impurities potentially harmful to humans such as anabasine, which were detected in most samples. Three different e-cigarette cartridges with the same label had markedly different amounts of nicotine (26.8 to 43.2 mcg /100 mL puff). There was bacterial (27%) and fungal (81%) contamination of single use and refillable products from 75 different manufacturers. Clearly quality control processes can be inconsistent or non-existent [41, 42]. Another study [43] demonstrated that all 122 the refill liquid samples analysed contained additives that are hazardous to human health, contrary to EU regulations which clearly prohibits ingredients posing a risk to human health in any form, with the exception of nicotine, being used in such liquids [44].

There are known effects of passive vaping, mandating the protection of non-vapers. In one study [45], nine volunteers (all occasional tobacco smokers) vaped nicotine-containing and nicotine-free devices to determine the effects on those nearby. In the gas phase, which was obviously inhaled, there were substantial increases in 1,2-propanediol, glycerine, nicotine, PM_2.5_, polyaromatic hydrocarbons (carcinogenic) and aluminium. Analysis of the urine of the passive vapers showed increased levels of nicotine, cotinine and 3-HPMA, a metabolite of acrolein.

In summary, e-liquids are unregulated and contain known carcinogens, as well as numerous unknown chemicals. Is there any plausible biological model whereby inhaling aerosols from these liquids is anything other than a BAD IDEA?

**Text Box.**

The seven undeniable facts about electronic cigarettes and vaping.

- Nicotine, which is found in most e-cigarettes, is a drug of addiction, and harmful to the foetus and child; even if e-cigarettes only contained nicotine, they should be banned
- If you do not know the contents of an e-liquid, how can you say it is safe? Especially when you do know that quality control is poor, and known carcinogens can be detected
- An extensive range of lung disease are caused by acute and short-term vaping, and these far exceed any acute toxicity of smoking cigarettes
- If the acute and short-term effects of vaping are worse than those of tobacco, how can we possibly rationally believe that the chronic toxicity of e-cigarettes is less than that of tobacco
- The toxic effects of vaping overlap with those of tobacco, but e-liquids have additional toxicity not shared with tobacco – e-liquids are not a diluted down version of tobacco
- E-cigarettes are being actively and deliberately marketed to young people; they will lead to a generation of nicotine addicts, which will be a catastrophe, and whether they are a gateway to smoking is irrelevant
- E-cigarettes are equivalent or inferior to standard smoking cessation strategies in all but a small majority of adult smokers. Generally, they are not being marketed as a smoking cessation aid.

## What is the public health issue about vaping?

The figures are frightening. The prevalence of current e-cigarette use in USA high school students rose from 1.5% in 2011 to 27.5% in 2019. The corresponding figures for USA middle school students were from 0.6% to 10.5% over the same time period. Even more worryingly, JUUL use among 18 to 20-year-old Americans rose from 11.9% in 2018 to 23.9% in 2019. These disturbing figures are mirrored elsewhere around the world [46-49]. The Industry would have us believe that CYP are vaping rather than smoking. This too is a lie. For decades [50], the prevalence of smoking dropped before vaping came on the scene, and has stayed the same while vaping use has climbed [51]. So, what is the attraction? Most common reasons given by adolescents and young adults are curiosity, flavouring or taste, and low perceived harm compared to other tobacco products, and trying to quit smoking is not the main reason.****Flavored e-cigarette use among young adult current users (18-24 years of age) exceeds that of older adult current users (25 years of age and older), and of CYP who have ever tried an e-cigarette, most used a flavoured product [52].

In a study of nearly 3,500 Californian high school students showed that electronic cigarette use in adolescents was associated with a higher risk of more frequent and heavy smoking 6-months later. A meta-analysis of more than 8000 American adolescents and young adults who were non-cigarette smokers at baseline showed that the probability of conventional cigarette smoking initiation was nearly four-fold greater than in non-users. A longitudinal study of American high school students found electronic cigarette use was associated with subsequent initiation of tobacco use, and the frequency of the use of both increased over time, consistent with nicotine addiction. Vaping is a one-way ticket for CYP to progress to cigarette smoking [53-55].

## Definitions: EVALI or EVALD

In 2019, numerous, previously healthy adolescents and young adults were hospitalised across the United States with severe respiratory illnesses related to “vaping”, what coined the term e-cigarette and vaping acute lung injury (EVALI) [56]. The diagnosis requires a history of e-cigarette, or vaping, product use in the 90-days before symptom onset. Most patients presented with various respiratory findings and pulmonary infiltrates on chest imaging, but often gastrointestinal, renal, and constitutional symptoms were described. EVALI is a diagnosis of exclusion, and alternative causes should be considered, including a negative viral assays with negative influenza and SARS-CoV-2 tests. 

Different lung pathologies have been described in people with EVALI, including acute eosinophilic pneumonia, organising pneumonia, lipoid pneumonia, and alveolar haemorrhage [57-61]. Eosinophilic pneumonia may be an early complication of vaping with an acute onset mandating ventilator support. Computed tomography (CT) shows septal thickening and pleural effusions. Bronchoalveolar lavage fluid (BALF) is eosinophilic, and the treatment is systemic corticosteroids. By contrast, organising pneumonia is usually seen after prolonged periods of vaping and can have a subacute onset. Mechanical ventilator support may be needed. BALF analyses are often nonspecific, high-resolution CT shows microlobular or centrilobular opacities, and the treatment is systemic corticosteroids. Lipoid pneumonia is another subacute complication of prolonged vaping, and symptoms may include fever and weight loss. Chest imaging shows a ‘crazy paving’ appearance, and BALF contains lipid-laden macrophages. Corticosteroids are often given, but are of uncertain value. Alveolar haemorrhage can be a complication of intense vaping. The person may present with haemoptysis, but it is not inevitable, and diffuse alveolar infiltrates typical of alveolar bleeding are seen on CT. Lavage fluid is positive for haemosiderin-laden macrophages. There is no proven treatment. Other complications associated with EVALI include air leaks which may be extensive [62], hypersensitivity pneumonitis, transient nodules, severe airflow obstruction, respiratory bronchiolitis associated interstitial lung disease, pleural effusion and upper airway damage [63].

For these reasons, the term, EVALI, may be misleading. Because there is more to the acute toxicity of vaping than acute lung injury, perhaps the term e-cigarette and vaping acute lung disease (EVALD) may be preferable. EVALD is not a single condition, but an umbrella term which covers multiple different pathologies. Furthermore, the requirement to eliminate other causes of diffuse alveolar damage is not straightforward [64, 65]. Those with pre-existing lung disease are surely not immune from e-cigarette toxicity; if a teenager with, for example, a surfactant protein C mutation deteriorates after vaping, is that EVALI/EVALD or an acute interstitial lung disease lung attack? The question is likely impossible to answer. During the current pandemic, it is clear that vaping, especially in combination with tobacco abuse, greatly increases the risk of coronavirus disease-19 (CoVID-19) infection, possibly via upregulation of the angiotensin converting enzyme (ACE)-2 receptor by nicotine [66]. Furthermore, a patient with an acute e-cigarette lung disease may require positive pressure ventilation, and suffer secondary volutrauma, nosocomial pneumonia and other iatrogenic complications, which may obscure the contribution of vaping. It may be worth breaking down the classification of EVALD as in the Table. Whether or not the concept of EVALD is thought useful or enters common usage, it would be a big mistake to assume that the toxic effects of vaping are confined to acute lung injury.

**Table.** Classification of EVALD. The diagnosis requires the presence of a pulmonary disease which may be related to e-cigarettes, together with an exposure history

Probable EVALD	No other underlying cause for the lung disease identified
Possible EVALD	Other at least potential aetiologies, such as pre-existing lung disease, iatrogenic complications of intensive care Acute lung injury especially, or other EVALD with no other aetiological explanation
(Hypothetically) passive EVALD	Exposure history is second hand; there are undoubted consequences of second hand vape exposure, unknown whether this includes EVALD

## What is the acute toxicity of e-cigarettes?

The most dramatic are burns and blast injuries from device malfunction, including a shattered mandible in one case [67, 68]. Much more serious is the epidemic of EVALI/EVALD, and its prevalence is rising [56, 69]. The tobacco industry has tried to obfuscate by pointing out that much EVALI/EVALD is related to cutting the e-liquid with cannabinoids. However, at least 20% of cases are not cannabinoid related, and the cannabinoid-related cases question the wisdom of allowing devices that can be abused in such a manner to be widely circulating in the community. 

The exact cause(s) of the lung pathologies in EVALI/EVALD is unknown. With so many potential candidate chemicals, it is likely to remain so for many conditions. However, vitamin E acetate has been found in BALF and implicated in some EVALI patients, causative in lipoid pneumonia. In a murine model, mice were exposed to propylene glycol-vegetable glycerine or vitamin E by inhalation, and vitamin E levels in treated mice had lavage levels similar to those found in human vapers. The pathology of human lipoid pneumonia was mimicked, with mononuclear cell inflammation and large numbers of lipid laden macrophages in BALF and lung histology samples [70]. 

**Practice point:** if a CYP presents with an unusual or atypical respiratory illness, consider if it may be due to vaping. Always take a vaping history as well as a smoking history

In summary, the acute toxicity of vaping is far greater than that of tobacco; so how is it possible to be complacent about long term toxicity? In the future, it is likely that further respiratory diseases will be discovered to be part of EVALI/EVALD.

## What is known about the chronic toxicity of e-cigarettes?

Information is limited for the simple reason that they have not been available for as long as tobacco. The first mass produced and marketed electronic cigarette was developed in China less than two decades ago, and later introduced to the Western marketplace. However, there are some pertinent facts:

1. It took decades before the fact that smoking caused lung cancer was determined by Sir Richard Doll, and even longer before everyone was convinced of the causal relationship.
2. During this period, the tobacco industry, those pillars of rectitude and transparency, and who now make vapes, deliberately tried to conceal facts and data and obscure the truth.
3. We are still making new discoveries about cigarette toxicity, years after the death of Dr. Doll.

Given these facts, how can anyone make statements, such as Public Health England assessment, that “e-cigarettes are 95% safer than tobacco”. It is an intellectually indefensible position, based on inadequate evidence.

## What have the laboratory studies taught us about e-cigarette toxicity?

A number of studies (below) have highlighted adverse effects of e-liquids, but it should be borne in mind that with such a multiplicity of different liquids, there can by definition be no standardised ecigarette model, a fact which the tobacco industry has used to try to discredit such efforts.

One siren cry of the tobacco industry is that e-cigarettes are less damaging than tobacco. While admitting that mice are not men, a murine emphysema study casts doubt on this [71]. Six week Sprague-Dawley rats were exposed to one of four conditions: room air (control), subcutaneous nicotine, e-cigarette vapour and cigarette smoke. Quantitative lung histology showed that all three interventions caused equally severe emphysema compared to controls; not very reassuring of the safety of e-cigarettes! There are concerning reports about the effects of vaping on the immune system. An *in vitro* study of alveolar macrophages showed that macrophage apoptosis and necrosis was increased by vape condensate [72]. Lower doses led to 50-fold increase in reactive oxygen species and inhibited macrophage phagocytosis. Given the pivotal role of alveolar macrophages in host defence, these data highlight a seriously worrying pro-inflammatory and immunosuppressive effect of vaping.

It is important to stress that the adverse effects of e-cigarettes, while overlapping with those of tobacco, have distinctive features. In a research study examining pulmonary effects in long-term smokers, vapers, and control subjects, 300 proteins were differentially expressed in the lungs of smokers and vapers, of which 113 were upregulated only in vapers. E-liquids entered cells and affected membrane fluidity and protein diffusion [73]. Another study evaluating the effects of vaping on the innate immune system used induced sputum in smokers, vapers, and normal subjects to study quantitative proteomics [74]. They found that vaping increased oxidative stress, and sputum elastase and matrix metalloproteinases. There were also increased neutrophil and NET-associated proteins and change in mucus composition (MUC5AC and 5B upregulation) [74]. Peripheral neutrophils showed increased NETosis in e-cigarette users. Again, although there were commonalities in the modulation of innate immune pathways between smokers and vapers, many pathways were only perturbed in vapers, not smokers.

## What should be our strategy to protect CYP?

To summarise the above findings, we do not know what is being inhaled with these nicotine delivery devices, but we do know the effects are not just a weakened version of tobacco; they have their own brand of harm. Furthermore, nicotine is a component of e-cigarettes, it is a drug of addiction and on its own is poisonous to the developing lung and elsewhere.

The Federation of International Respiratory Societies (FIRS) and the European Respiratory Society has led the way opposing vaping. This collaborative of nine international professional organizations, has made the following recommendations concerning electronic cigarettes in CYP [75, 76]. Electronic nicotine delivery systems should be considered tobacco products and regulated as such, including for taxation purposes. The sale of electronic cigarettes to CYP must be prohibited by all nations, and those bans must be enforced. All forms of promotion must be regulated, and all advertising in media (including social media) accessible to CYP should cease. Flavourings should be banned. And finally, regular surveillance should be performed to better understand the scope and health threat of tobacco products to CYP. To which we would add the need for severe penalties as enforcement for advertising to CYP. We insist on plain packaging with the health warning that the acute consequences of vaping may be life-threatening, and the long-term consequences are unknown. We advocate for a ban on vaping in all public places, because otherwise you have an army of unpaid vape-promotors.

We also have to be aware that the industry is deliberately marketing vaping to the young, and have indeed been charged as such by the US FDA, who regard vaping in children and young people as a serious epidemic. The use of flavourings, which many advocate banning, is clearly to make these devices more attractive to CYP [77]. Electronic cigarette marketing themes are similar those notorious in the past in cigarette advertising targeting youths, focused on freedom and glamour. Advertising tries to present a friendly face by marketing vaping products with a variety of unsubstantiated health and cessation messages, and have been advertised on both radio and television in many parts of the world. Social media platforms has become a prominent conduit for marketing and advertisement. In a 4-year study of nearly 250,000 Instagram posts, vaping hashtags were used 10,000 times more than FDA warnings, and indeed the warnings seemed counterproductive, prompting a surge in posts and likes about vapes. Pods which give a nicotine surge were posted very frequently, and worryingly, there were many under-age followers recorded [78]. Despite efforts restrict youth access, these products can be obtained by young people of any age in stores or over the internet [79-81]. The Industry would have us debate whether or not vaping is a gateway to smoking. Restrictions only work if they are enforced. A recent study showed that children aged 14 to 17 years successfully received deliveries of electronic cigarettes from 32% of purchase attempts, using credit cards or electronic checks. After excluding the orders that failed for reasons unrelated to age verification (e.g. failed payment processing), teenagers were always successful in buying from vendors [82]. We must do more. To these authors, it is an irrelevant question; vaping is a dangerous habit in its own right, whether or not it is a stepping stone to vaping, and must be vigorously countered. 

The next distracting tactic the tobacco industry has deployed is that vaping is a gateway to smoking cessation. Their own advertising tactics undermine this argument. We invite our readers to compare the advertising for established cessation aids, such as nicotine containing gum, with that of e-cigarettes. Superficially, there are supportive data. In one study [83], 886 smokers were randomised to either conventional nicotine replacement or e-cigarettes. In both cases, patients were given a choice of options. The one year abstinence rate was 18.0% in the e-cigarette group, compared with 9.9% nicotine replacement (RR 1.83, CI 1.3-2.58). However, closer scrutiny renders these conclusions less impressive. Firstly, 80% assigned to e-cigarettes were still using them after a year, in other words, had exchanged one dangerous habit for another. The accompanying editorial [84] also pointed out that the results in both limbs were inferior to those reported with nicotine replacement therapy and buprion (25-26% quit rate at 6 months, 20% 1 year abstinence rates) and with varenicycline (27% at 24 weeks). In a pragmatic trial in 6006 smokers, randomisation was to usual care or usual care plus one of (a) free nicotine replacement; (b) free e-cigarettes; and two limbs with different financial incentives [85]. The result showed that money talks! The 6-month abstinence rates were much higher in the groups offered financial rewards. Finally, the UK National Institute for Clinical Excellence (NICE) does not recommend e-cigarettes as an aid to smoking cessation. We concede that there may be a few hard-core smokers for whom the only hope of possible harm reduction is vaping, but these are a minority and not at the cost of child health.

## Summary and conclusions

The siren and duplicitous cry of the tobacco and vaping industry, those pillars of transparency and rectitude, is that we need more studies to evaluate whether vaping is safe. This is a lie. The default position, the line we must hold, is that it is not the job of the medical and scientific professionals to determine if vaping is safe. It is the job of the protagonists of this dangerous habit to show that it is safe, both short- and long-term. It took decades for the long-term harm of tobacco to be detected; when will we know about vaping? And if acute toxicity of vaping is worse, how can we be complacent about the long term? We know that inhaling different chemicals can cause unexpected damage; so why would we be surprised to find that the same is true of vaping? Vaping is still largely unregulated, not exactly a recommendation that it is safe. We need to call on legislatures to stamp down on this new subversive attempt by the industry, and at the very least, treat electronic and combustible cigarettes the same way. The data showing e-cigarettes are an effective smoking cessation aid is underwhelming, while evidence that they are being marketed at children overwhelming. Is there a biologically plausible model whereby vaping is good?

Two final points. As a practice point, if you see a respiratory oddity, ask if could this be due to vaping? And lastly, as the old Italian proverb states, “Fool me once, shame on you; fool me twice, shame on me”. The Industry fooled us once over tobacco - will we let them fool us a second time over vapes? 

***Andrew Bush is an Emeritus NIHR Senior Investigator. Thomas Ferkol is past President of the American Thoracic Society and Forum of International Respiratory Societies. Arūnas Valiulis is Planning Committee of Global Alliance Against Chronic Respiratory Diseases (WHO GARD) member and leader of project No. P-MIP-20-404 of Research Council of Lithuania.***
